# A Scoping Review on the Incidence, Risk Factors, and Outcomes of Proximal Neck Dilatation after Standard and Complex Endovascular Repair for Abdominal Aortic Aneurysms

**DOI:** 10.3390/jcm12062324

**Published:** 2023-03-16

**Authors:** Luca Mezzetto, Mario D’Oria, Sandro Lepidi, Davide Mastrorilli, Cristiano Calvagna, Silvia Bassini, Jacopo Taglialavoro, Salvatore Bruno, Gian Franco Veraldi

**Affiliations:** 1 Unit of Vascular Surgery, Integrated University Hospital and Trust of Verona, Piazzale A. Stefani 1, 37124 Verona, Italy; 2 Division of Vascular and Endovascular Surgery, Cardiovascular Department, University Hospital of Trieste ASUGI, 34129 Trieste, Italy

**Keywords:** aortic disease, aortic aneurysm, endovascular repair, aortic neck, scoping review, outcomes

## Abstract

**Background:** To define proximal neck dilation (PND) after standard endovascular aneurysm repair (EVAR) and fenestrated EVAR (FEVAR), determining: incidence and risk factors; evidence base that links PND to outcomes of patients; recurring themes or gaps in the literature. **Methods:** We performed a scoping review and included only full-text English articles with follow-up focusing on PND in patients undergoing EVAR or FEVAR, published between 2000 and 2022. The following PICO question was used to build the search equation: in patients with abdominal-aortic-aneurysm (AAA) (Population) undergoing endovascular repair (Intervention), what are the incidence, risk factors and prognosis of radiologically defined PND (Comparison) on short-term and long-term outcomes (Outcomes)? **Results:** 15 articles were included after review. Measurement protocols for proximal aortic neck (PAN) varied among individual studies and the definition of PND resulted as heterogeneous. Rate of patients with a PND ranged between 0% and 41%. Large proximal neck (>28 mm) and excessive graft sizing (30%) were predictors for PND. New endografts with low outward radial forces and FEVAR seemed to be protective. Surgical conversion was the definitive option in the case of patients unfit for other endovascular treatments. **Conclusions:** PND is a frequent finding after EVAR and FEVAR. Excessive graft oversizing and large baseline PAN were predictors of neck enlargement, independently by the type of standard endograft used. FEVAR may be considered protective against complications, together with endografts using low outward radial forces. Lifelong radiological follow-up is mandatory.

## 1. Introduction

Despite favorable rates of early morbidity and mortality of endovascular aneurysm repair (EVAR) in patients with abdominal aortic aneurysms (AAA), the need for late reinterventions still represents a considerable issue [1,2]. Careful evaluation of the baseline anatomical characteristics, including proximal and distal neck morphology, endograft selection (e.g., standard EVAR with infrarenal vs. suprarenal fixation, fenestrated endovascular aneurysm repair—FEVAR—for complex AAA morphology, choice of different oversizing ratios, etc.), as well as lifelong follow-up are essential in the prevention, early detection, and prompt management of such complications. Freedom from loss of proximal sealing zone is the key for durability of this minimally invasive approach and it is guaranteed by an adequate connection between the proximal aortic wall and the stent graft. Continuous aortic remodeling after EVAR has been largely described, including angle modifications and proximal neck dilatation (PND) [3,4]. Several observational clinical studies have explored PND, with contradictory results regarding its incidence and its clinical impact [5,6,7]. The primary objectives of this scoping review were: (i) to define PND after EVAR and FEVAR, (ii) determine its incidence and risk factors, (iii) identify the evidence base that links PND to outcomes of patients undergoing endovascular treatment, and (iv) analyze recurring themes or gaps in the literature to guide future research.

## 2. Methods

### 2.1. Study Design

**Figure 1:**
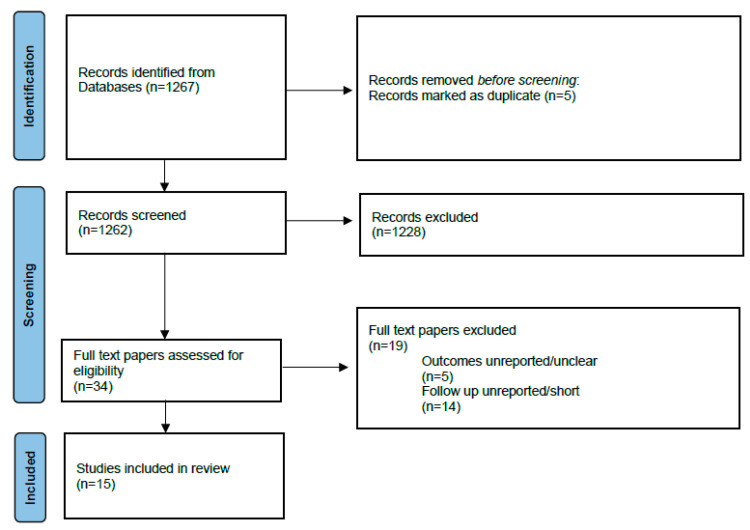
PRISMA flowchart of study selection.

A scoping review following the PRISMA Protocols Extension for Scoping Reviews was performed [6] (Figure 1).

Available full-text studies published in the English language in PubMed, Cochrane, Web of Science and EMBASE databases after 2000 (last queried, 30 June 2022) were systematically reviewed and analyzed. Reference lists from all included manuscripts were also manually screened and included, if necessary. The following PICO question was used to build the search equation: in patients with AAA (Population) undergoing endovascular repair (Intervention), what are the incidence, risk factors, and prognosis of radiologically defined PND (Comparison) on short-term and long-term outcomes (Outcomes)?

Only full-text articles focusing on proximal neck dilation (PND) in patients undergoing standard endovascular abdominal aortic repair (EVAR) or fenestrated endovascular aneurism repair (FEVAR) for the treatment of infrarenal, pararenal, and juxtarenal aneurysm were included. Only reports with long-term follow-up (maximum follow-up of at least 3 years) were considered for the analysis. Any type of diagnosis and indications for treatment, when reported by authors, were included in the analysis. 

Search terms were: ‘endovascular treatment’ OR ‘evar’ OR ‘fenestrated’ OR ‘FEVAR’ AND ‘neck dilation’ OR ‘Neck enlargement’ Or ‘juxtarenal aneurysm’ OR ‘pararenal aneurysm’, in various combinations.

Identified titles and abstracts were reviewed by two independent authors (L.M., M.D.), and any areas of disagreement were discussed with a third author (D.M.). Duplicate copies of articles were identified and removed. Manuscripts were also excluded if they were case reports, letters, editorials, commentaries or were written in a language other than English. Papers reporting unclear method of aortic neck measurement and reporting data of FEVAR after failure EVAR were also excluded.

### 2.2. Data Extraction

The following variables were extracted and reported: year and country of study, study design, patient number, demographics, proximal neck measurements, intervention type, endograft brand, as well as short-term and long-term outcomes (with follow-up time), defined as the onset of type IA endoleak, graft migration and reinterventions. Data were reported as descriptive narrative or tables, without any statistical analysis nor quality assessment of the included papers, in accordance with the PRISMA guidelines for scoping reviews.

## 3. Results

### 3.1. Literature Search

After reviewing full texts, 34 articles were considered as relevant and 15 were finally included in the scoping review (Figure 1). Briefly, 11 papers focused on EVAR [4,5,6,7,8,9,10,11,12,13,14], 3 on FEVAR [15,16,17], and 1 on EVAR and FEVAR [18], including elective and non-elective presentations. Overall, we included 2574 subjects of which 2361 received EVAR and 213 received FEVAR, respectively. Most articles were retrospective in design and only two papers reported data from a prospective enrollment. There were no randomized controlled trials available for inclusion (Table 1). 

Results from different standard aortic endografts were reported in EVAR manuscripts while custom-made Cook and physician-modified endografts (PME) were included in FEVAR group. Baseline characteristics of the included patients were reported in Table 2.

Although all the included manuscripts clearly reported their measurement protocol for the diameter of PAN, the methods varied between the individual studies (Table 3). 

The perpendicular plane to the longitudinal outer-to-outer axis at the lowest renal artery represented the commonest and more intuitive approach [4,5,6,8,11,12,13,14,17]. Several authors extended the measurement 5 mm–20 mm below the renal arteries level or to the suprarenal aorta, at the level of the superior mesenteric artery (SMA) or celiac artery (CA). Measurements at the level at the proximal edge of the stent graft and of other fixed graft markers were also described. Crude values at the level of interest [8,11,13,14,16,17] or an average between different levels [4,5,6,7,10,15,18] were reported to describe the metric details of the PAN.

Among EVAR patients, only suprarenal fixation was reported in 4 manuscripts [4,9,12,14]. Both suprarenal and infrarenal fixations were included in the other available studies. The sole paper comparing outcomes of FEVAR vs. EVAR, included patients with suprarenal and infrarenal fixation endografts. Complete adherence to Instructions for Use (IFU) of aortic endografts was reported in only 5 papers [4,11,12,13,14].

Graft oversizing was reported as mean (±SD) or as range of values and it varied between 10% and 28% in EVAR and between 9.9% and 22% in FEVAR procedures.

Characteristics of PAN also differed significantly among the included studies (Table 3). For authors referring the measurement at the lowest renal artery, mean neck diameter ranged between 23.8 mm and 25.5 mm. In the case of measurement extension to the suprarenal aorta, the mean neck diameter resulted to be slightly larger, ranging between 25.7 mm and 29.7 mm, while in FEVAR the mean suprarenal and infrarenal diameter was 25.5–26.6 mm and 23.5–25.7 mm, respectively.

### 3.2. Definition of PND

According to the available literature, the definition of PND resulted as highly heterogeneous. Where reported, a high grade of intra-observer or intra-observed agreement was documented [4,7,9]. Five studies defined PND as an enlargement of at least 3 mm from the baseline, while only one study considered 2 mm as significant. A relative increase of +10% and +15%% from the baseline was defined in two studies. In the remaining papers, a quantitative change was reported without defining a clear cut-off. 

Overall, thin-slice computed tomography angiography (CTA) images were compared to slices at same level of preoperative or one-month CTA. No other radiological techniques (such as ultrasound or magnetic resonance) were described for PND measurement.

### 3.3. Incidence of PND and Risk Factors

#### 3.3.1. EVAR Group

In the EVAR group, 8 studies reported outcomes after 5 years from the index procedure [4,6,7,8,10,11,12,14]. Incidence of PND depended upon the methods of measurement and the standards of reporting. Among the authors referring to PND as an increase of at least 3 mm or more from the baseline, the rate of patients with a significant change of anatomy ranged between 4.2% and up to 35%, although De Donato et al. did not report any significant change (≥2 mm) of PAN during follow-up [9]. Torsello et al. documented an increase >15% from the baseline in 41% of the included patients with an average growth of 4.5 mm under the lowest renal artery [14]. Continuous growth of the infrarenal proximal neck (0.17–1.0 mm/year) was described in three studies, particularly during the early period from the procedure [4,5,13]. Behavior of suprarenal aorta after EVAR remained debated even if it seemed to be less prone to dilation during follow-up, after both suprarenal or infrarenal fixation [2,7]. 

Although PND may be unrelated with type of standard endograft, planning of the procedure is crucial to reduce the risk of neck complications as an excessive graft sizing, particularly >30 %, has been well described as a predictor of neck dilation in several studies (OR 1.07, 95% CI 1.04–1.10, *p* < 0.001) [7,14,16]. Also, extremely low rates of PND were reported in two studies regarding the Ovation Trivascular, an endograft with low outward force at the sealing zone, strengthening this hypothesis and emphasizing the importance of graft selection during preoperative assessment [9,12]. 

Preoperative evaluation of proximal neck diameter is also essential to reduce the risk of PND after EVAR. Indeed, Oliveira et al. revealed that among 132 patients, those 21 (15.9%) with baseline neck diameters ≥ 30 mm reached the 90% endograft expansion threshold earlier (median 2.8 vs. 4.3 years, *p* = 0.026) [7]. Similar results were observed by Gargiulo et al. in patients with aortic diameter ≥ 28 mm [10]. The role of other morphological characteristics at the level of proximal neck, such as calcifications and thrombus, has not been extensively defined in the included manuscripts.

#### 3.3.2. FEVAR Group

In the FEVAR group, all studies reported outcomes after 3 years from the index procedure with only one study that extended data after 4 years [18]. Four studies analyzed PAN after FEVAR [15,16,17,18]. Among 84 patients with available CTA in the study by Rastogi et al., the median PND was 9.5% (IQR, 4.6–18.3%), and PND of >10% and >20% at the level of the first covered stent was found in 47.6% and in 20.2%, respectively. Similar results were obtained by Tran et al., who experienced 74.4% of diameter growth at infrarenal level and found that both larger preoperative aortic seal diameter (+0.41 mm of dilation per 1 mm increase in preoperative aortic diameter; *p* = 0.003) and more aggressive graft oversizing (+1.34 mm per 10% increase in oversizing; *p* = 0.020) were both predictors of future neck dilation. Increasing device oversizing relative to the native visceral aorta was a predictor of postoperative neck diameter growth (1.34 mm per 10% increase in oversizing; *p* = 0.02); meanwhile, a higher number of fenestration and increasing proximal seal length resulted to be protective (−1.82 mm per 10 mm increase in seal length; *p* = 0.016) [16]. 

At 3 and 5 years, freedom from excessive dilatation was 92.3% (95% CI, 85.9–99.1%) and 81.6% (95% CI, 69.7–95.5%), respectively [15]. When compared with EVAR subgroups, FEVAR demonstrated the least neck dilation and the greatest degree of sac regression (FEVAR, 15.9%; Endurant, 6.5%; Excluder, 3.0%; Zenith, 3.4%; *p* = 0.009) [18].

### 3.4. Impact of PND on Outcomes (Survival, Reinterventions, Endoleaks, Sac Increase)

#### 3.4.1. EVAR Group

The relationship between the neck growth and the onset of any neck complication (migration, type IA endoleak, and reinterventions) remained controversial. In one study including 60 patients, there was no relation between PAN dilation and either type IA endoleak or sac diameter changes during 5 years (r = 0.17; *p* = 0.991) [14]. Other authors reported different outcomes and demonstrated that excessive neck dilatation was associated with type 1A endoleaks (HR 3.3, 95% CI 1.1–9.7), endograft migration (HR 3.1, 95% CI 1.4–6.9) and neck-related adverse events overall (HR 2.6, 95% CI 1.3–5.2), but not with AAA sac growth [7]. Gargiulo et al. documented the threat of PND in 118 patients who experienced 12% of type IA endoleak and required surgical conversion in 5.9% of cases. Survival at 3 years and 5 years was 89% (standard error [SE], 0.03) and 70% (SE, 0.05), respectively; on multivariate logistic regression, there were no risk factors associated with overall mortality [10].

Different therapeutical approaches have been described for patients with PND and neck complications: the most frequent treatment was proximal extension with cuff, with or without fenestrations. Alternative endovascular approaches were angioplasty, embolization, and chimney EVAR. Surgical conversion was the unique open approach in the case of sealing failure. 

Clinical outcomes appeared particularly favorable in cases of endograft with low outward radial force [9,12]. The rate of type IA endoleak ranged between 1.2% and 3.7%, and only one endovascular reintervention for proximal neck complication was necessary among 399 included patients. The 5-year freedom from all-cause mortality was 74.9%, and freedom from aneurysm-related mortality was 99.3%.

#### 3.4.2. FEVAR Group

Rates of type IA endoleaks were very low among the included patients (3/213; 1.4%) and no conversions were described during the available follow-up. Compared with the EVAR group, FEVAR patients were more likely to demonstrate sac regression (≥5 mm) at both 1-year and the last available follow-up (52.38% vs. 27.53% [*p* = 0.034]; and 63.33% vs. 42.22% [*p* = 0.036], respectively) [18]. Also, PND was not associated with type IA endoleak (*p* = 0.256), renal stenosis or occlusion (*p* = 0.672), and reinterventions (*p* = 0.99) [16]. No differences were found in the incidence of endoleaks, migrations, and reinterventions between patients with and without infrarenal neck dilation [17]. Clinical outcomes of all patients in the studies are summarized in Table 4. 

## 4. Discussion

This scoping review confirmed that PND is a frequent finding during long-term follow-up after endovascular repair of AAA: in particular, up to 41% of EVAR may reveal a significant enlargement of PAN, as confirmed on contrast-enhanced cross-sectional imaging. These results are slightly different from previous evidence that reported an incidence after EVAR of approximately 23%, probably because of our longer follow-up and the inclusion of FEVAR cases [19,20]. The mechanism resulting in PND is likely multifactorial and mainly depends on the combination of several factors including natural history of degenerative aortic disease and mechanical effects of endografts into the aortic lumen [21]. 

Growth of the proximal neck has been described in two phases: the first one, usually more rapid and depending on stent-graft oversizing (which is an indirect measure of the chronic outward radial force that is exerted on the aortic wall), occurs during the early postoperative period; the second one, probably related to aortic wall degeneration, is a continuous and slower neck dilation that may be observed during long-term follow-up, even in patients with AAA sac shrinkage [4,22,23]. The causative role of endograft sizing on neck changes has been widely discussed: in a recent analysis on 154 patients, oversizing <10% had less impact on the suprarenal aorta than >15% oversizing at 4 years (0.41 mm, CI: −0.31 to −1.14 vs. 3.26 mm, CI: 1.63–4.88, *p* < 0.001); however, it was also noted that oversizing had a more pronounced effect on the infrarenal aorta: 3.01 mm, CI: 2.18–3.83; 5.95 mm, CI: 3.26–8.64; and 5.05 mm, CI: 3.41–6.69 for <10%, 10–15%, and >15% oversizing at four years, respectively [24]. Although a positive correlation between oversize and neck dilation was reported by several authors, it was not confirmed in another more recent analysis [4,25,26]. However, PND does not seem to be associated with any particular type of endograft; indeed, in the study by Deltomme et al., four types of contemporary aortic endografts were all associated with progressive dilatation of the PAN over a time interval of five years and were shown to be potentially associated with the development of type 1A endoleaks [8].

Suprarenal and infrarenal aorta appear to have differing behavior, and the first one seems to be less prone to dilation over time; this is probably the reason for the similar outcomes among grafts with different levels of fixation and the protective role of FEVAR in juxtarenal and pararenal aneurysms [8,27]. This may be explained by the fact that mechanical stress interacts with the patient’s aorta biomechanical properties that are influenced also by genetic and histopathologic factors. For instance, recent evidence has shown that matrix metalloproteinases, long known for their association with AAA incidence, are also linked to AAA sac behavior after EVAR [28]. Therefore, it would be reasonable to assume that they can also play an important role in the occurrence of PND over time and explain some of the differences observed.

Various levels of measurement (lowest renal artery, under lowest renal artery, superior mesenteric artery, fixed stent-graft markers) and various definitions (2 mm, 3 mm, 15–20% from baseline) have been proposed to define PND, but general agreement over a standard method of evaluation and a uniform threshold over which dilation should be considered relevant is still lacking. These considerations may justify the relatively low incidence of type IA endoleak and graft migration that were observed in the cohort of patients included in this analysis. Indeed, the efficacy of proximal sealing depends on the combination of several patients-related and graft-related factors, such as neck shape, angulations, calcifications, endoprosthesis fixation (barbs, bare metal stents), and the sole occurrence of 2–3 mm PAN growth from the baseline may be insufficient to cause any clinically relevant proximal neck complications [29,30,31]. Although this may represent a potential methodological bias, a continuous growth of the PAN has been well documented in most available literature and lifelong follow-up is recommended both in EVAR and FEVAR procedures, especially when hostile anatomical features of the PAN are present [32], as also suggested by current clinical practice guidelines [33]. Together with other baseline anatomical characteristics (such as distal aortic diameter and psoas muscle trophism [34,35,36]), the mean diameter of infrarenal sealing required careful attention in EVAR planning and graft selection. According to a recent systematic review of over 7448 patients, the rates of type IA endoleaks, reinterventions, and migrations were all significantly higher in the wide-neck (>25mm) patient group compared with patients with normal aortic neck size through follow-up [37]. Indeed, it is well known that achieving a seal zone in a morphologically infrarenal hostile neck will make the repair more prone to lower durability over time, especially for patients with larger proximal requiring higher oversize. Fenestrated-branched EVAR (F-B/EVAR) moves the proximal zone above the renal-visceral vessel where the native aorta is less prone to dilation and it may represent a feasible and safe alternative to EVAR in the case of hostile neck, especially for patients unfit for open surgical repair or for those with long life expectancy [38]. According to the outcomes of high-volume aortic centers, F-B/EVAR may be also proposed as the first-line approach in the case of proximal complication after EVAR, with satisfactory clinical outcomes comparable with patients undergoing treatment for native aneurysm [39,40].

The use of endografts based on an alternative sealing concept that uses a polymer-filled sealing ring and provides uniform, non-expansive, continuous wall apposition thus reducing the outward radial force, has been proposed as an alternative to standard EVAR. A multicenter observational study of 161 Ovation endografts reported no neck dilation after a minimum follow-up of 24 months. Similarly, Mathlouthi et al. estimated 97.7%, 96%, and 93.6% freedom from PND at 1, 3, and 5 years, respectively, without any case of endograft migration. However, further evidence is needed before use of this device may be warranted with the sole purpose of reducing the incidence of PND, as several factors may come into play when choosing the optimal endograft that will fit each individual patient’s anatomy.

Also, adjunctive procedures to standard EVAR may be useful to prevent PND and other proximal neck complications. An observational study comparing patients with hostile PAN treated with standard EVAR vs. endosutured aneurysm repair (ESAR) concluded that the use of EndoAnchors increased the rate of sac regression (*p* = 0.003) and reduced the number of type IA endoleaks (*p* = NS) [41]. However, this study did not provide any direct measurement of PAN evolution and/or PND; thus, any inferences as to whether the use of EndoAnchors may halt the process leading to neck enlargement remain purely speculative at this time. Nonetheless, Tassiopoulos et al. described results after 1 year of follow-up in 257 consecutive patients who underwent EVAR with Heli-Fx and suggested that EndoAnchors may be protective against PND, together with smaller baseline neck diameter and suprarenal fixation [42]. Larger cohorts of patients and longer follow-up are necessary to confirm the protective role of these technologies and to justify their wider use in patients with hostile PAN.

### Study Limitations

Given the scoping nature of this review, there is an element of selection bias in the identification of articles for inclusion. Relevant articles may also have been missed using the search parameters and literature search. This review presents heterogenous study designs and methods that hindered our ability to perform a formal meta-analysis. Furthermore, there were a variety of definitions used for the postoperative outcomes assessed as well as the length of follow-up for the included studies; in particular, a high grade of heterogeneity was observed between studies in measuring PAN and defining PND. This makes it difficult to generalize findings to all patients undergoing EVAR and FEVAR. Finally, the rate of procedures performed inside Instructions for Use (IFU) was rarely specified, and this may be a possible bias for clinical evaluation during long-term follow-up

## 5. Conclusions

According to the available English literature included, a relatively high occurrence of PND may be revealed after both EVAR and FEVAR, although this is not strictly related to other proximal neck complications. Excessive graft oversizing and large baseline PAN appeared to be predictors of neck enlargement, independently of the type of endograft used in standard EVAR. Thanks to the proximal extension of the sealing zone, FEVAR may be considered protective against complications, together with new-generation endografts using low outward radial forces. Lifelong radiological follow-up is mandatory, even in patients with sac shrinkage.

## Figures and Tables

**Table 1:** Characteristics of included manuscripts.

Author	Year of Publication	Country of Corresponding Author	Type of Study	Period of Enrollment	Type of Procedure	N. Patients	Type of Endograft
Kaladiji et al. [11]	2012	France	Retrospective, Single center	2003–2007	EVAR ^a^	61	Talent, Medtronic (31) Zenith, Cook (23) Excluder, Gore (6) Anaconda, Vascutek (1)
Savlovskis et al. [13]	2015	USA	Retrospective, Multicenter	2008–2010	EVAR	105	Endurant Medtronic (56) Nellix, Endologix (49)
Tsilimparis et al. [4]	2015	Germany	Retrospective, Multicenter	Ongoing	EVAR	736	Zenith, Cook
de Donato et al. [9]	2016	Italy	Retrospective, Multicenter	2010–2012	EVAR	161	Ovation, Endologix
Kret et al. [6]	2016	USA	Retrospective, Single center	2008–2014	EVAR	86	Zenith, Cook (26) Excluder, Gore (26) Endurant, Medtronic (22) Powerlink, Endologix (10) Ovation, Trivascular (2)
Gargiulo et al. [10]	2017	Italy	Retrospective, Multicenter	2009–2012	EVAR	118	Zenith, Cook (74) Endurant, Medtronic (28) Anaconda, Vascutek (9) Excluder, Gore (6) Ovation, Trivascular (1)
Vukovic et al. [5]	2018	Canada	Retrospective, Single center	2005–2014	EVAR	126	Anaconda (Vascutek, Inchinnan, United Kingdom)
Torsello et al. [14]	2019	Germany	Prospective, Multicenter	2010–2011	EVAR	60	Incraft
Deltomme et al. [8]	2021	Belgium	Retrospective, Single center	2007–2015	EVAR	120	Zenith, Cook (30) Excluder, Gore (30) Endurant, Medtronic (30) Ovation, Trivascular (30)
Mathlouthi et al. [12]	2021	USA	Prospective, Multicenter	2009–2012	EVAR	238	Ovation, Trivascular
Oliveira et al. [7]	2021	Portugal	Retrospective, Single center	2000–2015	EVAR	460	Endurant, Medtronic (243) Excluder, Gore (181) Talent, Medtronic (13) Zenith, Cook (6) Others (17)
Tran et al. [16]	2021	USA	Retrospective, Single center	2012–2018	FEVAR ^b^	43	ZFEN, Cook
Zettervall et al. [17]	2021	USA	Retrospective, Multicenter	ongoing	FEVAR	56	Zenith, Cook (23); Endurant, Medtronic (4); Treo, Bolton (1)
Teter et al. [18]	2022	USA	Retrospective, Multicenter	2011–2019	FEVAR	120	ZFEN, Cook (30); Endurant, Medtronic (30), Excluder, Gore (30), Zenith, Cook (30)
Rastogi et al. [15]	2022	Netherlands	Retrospective, Multicenter	2008–2018	FEVAR	84	ZFEN, Cook

^a^ EVAR: endovascular aneurysm repair; ^b^ FEVAR: fenestrated endovascular aneurysm repair.

**Table 2:** Patients characteristics of the included manuscripts.

Author	Age, Years	Male	Arterial Hypertension	Diabetes Mellitus	Respiratory Disease	Chronic Kidney Disease	Cardiac Disease	Aneurysm Diameter, mm
Kaladiji et al. [11]	74.6	57 (93.4%)	NA	5 (8.2%)	2 (3.3%)	1 (1.6%, end-stage)	26 (42.6%)	NA
Savlovskis et al. [13]	71,9	84 (80%)	NA	NA	NA	NA	NA	54.5 (50.5–60.0)
Tsilimparis et al. [4]	NA	NA	NA	NA	NA	NA	NA	55.4 (54.8–56.0)
de Donato et al. [9]	75.2	148 (92%)	94 (62%)	35 (22%)	NA	3 (2%)	57 (35%)	57.7
Kret et al. [6]	75.6	74 (86.1%)	73 (86,1%)	15 (17.4%)	18 (20.9%)	NA	43 (50%)	57.1 (42–82)
Gargiulo et al. [10]	73.9	NA (91%)	101 (86%)	22 (19%)	43 (36%)	30 (25%)	47 (40%)	60.8 (51–100)
Vukovic et al. [5]	70	96 (76%)	112 (89%)	26 (21%)	13 (10%)	32 (25%)	66 (52%)	NA
Torsello et al. [14]	74	57 (95%)	NA	NA	NA	NA	NA	52.6
Deltomme et al. [8]	72.7	112 (93%)	84 (70%)	16 (13.3%)	NA	51 (42.5%)	40 (33.3%)	63.5
Mathlouthi et al. [12]	73.3	193 (81%)	205 (86.1%)	54 (22.6%)	66 (27.7)	32 (13.4%)	108 (75.6%)	54 ± 8
Oliveira et al. [7]	73.1	408 (88.6%)	330 (71.7%)	78 (16.9%)	68 (14.7%)	101 (21.9%)	78 (16.9%)	59.0 (54.0–67.0)
Tran et al. [16]	72	37 (86%)	NA	NA	NA	NA	NA	61.9
Zettervall et al. [17]	74.5	44 (78.5%)	NA	12 (21.4%)	35 (62.5%)	NA	25 (44.6%)	62 (58–64)
Teter et al. [18]	73.5	NA	26 (86%)	4 (13.3%)	6 (20%)	NA	12 (40%)	NA
Rastogi et al. [15]	73	77 (91)	65 (81)	11 (13.4)	29 (36.3)	25 (29)	NA	60.1

Measurement of proximal aortic neck (PAN) and Procedural details.

**Table 3:** Anatomical and technical details.

Author	Level of Neck Measurement	Neck Dilation, Cut-Off	Neck Length, mm	Neck Diameter, mm	Graft Oversizing	Suprarenal Fixation	Infrarenal Fixation	Inside IFU
Kaladiji et al. [11]	Infrarenal aorta (D1a), 15 mm below the lowest RA ^a^ (D1b), the origin of the aneurysm (D1c)	≥3 mm	NA	D1a: 23.9 ± 3.3 D1b: 24.3 ± 3.9 D1c: 25 ± 4	16% (± 9%)	55 (90.1%)	6 (9.9%)	61 (100%)
Savlovskis et al. [13]	(1) below the lowermost RA (2) at the proximal end of the stent structure (3) 5 mm below the level of the proximal end of the stent	any variation	25.6 (19.9–31.4)	Level 1: 25.5 (23.9–26.5) Level 2: 25.4 (24.2–26.8) Level 3: 25.5 (24.3–26.7)	12.8% (±2.7%)	56 (53.3%)	49 (46.6%)	105 (100%)
Tsilimparis et al. [4]	lowest RA	any variation	NA	23.8 (23.6–24.1)	19% (±8%)	736 (100%)	0	736 (100%)
de Donato et al. [9]	Zone A: from upper limit of suprarenal stent to a tangent horizontal plane at the lowermost RA Zone B: from the lowermost RA to the first ring; Zone C: the first polymer-filled ring.	≥2 mm	NA	NA	10–20%	161 (100%)	0	NA
Kret et al. [6]	lowest RA and 10 mm below the lowest RA ^a^.	any variation	NA	24.5 (17.7–36.7)	13.6% (± 11.5%)	50 (58.1%)	36 (41.8%)	73 (84.8%)
Gargiulo et al. [10]	1 cm below the lowest RA (D1), at the level of each RA (D2, D3), at the level of the SMA ^b^ (D4), and at the level of the celiac trunk (D5).	≥3 mm	23.5 (10–37)	29.7 (28–36)	17% (± 9%)	102 (86.4%)	16 (13.4%)	94 (79.6%)
Vukovic et al. [5]	1 cm above and at the level of RA	any variation	>15	22 (21–24)	17% (9–26%)	0	126 (100%)	NA
Torsello et al. [14]	at lowest RA and 15 mm below the lowest RA	≥15% from baseline	NA	Lowest RA: 22.3 (17–29.5) Below RA: 23.0 (18–29.1)	NA	60 (100%)	0	60 (100%)
Deltomme et al. [8]	outer diameter at SMA and lowest RA	any variation	NA	SMA level: 25.7 (18.9–34); RA level: 24.3 (17.5–33)	10–20%	90 (75%)	30 (25%)	NA
Mathlouthi et al. [12]	lowest RA	≥3 mm	22 ± 12	22.4 ± 3	NA	238 (100%)	0	238 (100%)
Oliveira et al. [7]	start of proximal covered stent and lowest RA	any variation	28.0 (20.0–40.0)	24.0 (22.0–26.0)	20.0% (13.6–28.0%)	278 (60.4%)	182 (39.6%)	NA
Tran et al. [16]	A, top of the fixation struts; B, middle of the fixation struts; C, top of the graft fabric at the level of the gold marker; D, middle of the first seal stent; E, bottom of the first seal stent; F, middle of the second seal stent; and G, bottom of the second seal stent	≥3 mm	NA	Suprarenal: 25.8 Infrarenal: 25.7	19.6% (±5.5%)	43 (100%)	0	NA
Zettervall et al. [17]	at SMA and lowest RA	≥3 mm	NA	SMA level: 25.5 RA level: 25.4	SMA level: 16.5% (9.9–22%) RA level: 15% (11–20%)	56 (100%)	0	NA
Teter et al. [18]	The aortic neck and visceral aorta were divided into 5 mm segments ranging from 20 mm above the lowest RA to 20 mm below the lowest RA.	Any variation	NA	FEVAR ^c^: 26.6 (±4.0) EVAR ^d^: 23.5 (± 2.9)	FEVAR: 14.4% EVAR: 18.3%	90 (75%)	30 (25%)	NA
Rastogi et al. [15]	at both the start of the covered stent and at the bottom of the scallop and averaged as well	≥10% of baseline	29.5	25.4 ± 2.7	20.1% (± 9.1%)	84 (100%)	0	NA

^a^ RA: renal artery; ^b^ SMA: superior mesenteric artery; ^c^ FEVAR: fenestrated endovascular aortic repair; ^d^ EVAR: endovascular aortic repair.

**Table 4:** Clinical outcomes.

Author	F-Up, Months	Neck Dilation	Graft Migration	TIaEL	AAA-Related Reintervention	Neck-Related Reintervention	Type of Reinterventions
Kaladiji et al. [11]	39 (24–84)	Dilation: 3.7 mm ± 2.8 for D1a; 4.4 mm ± 2.5 mm for D1b; 4.4 mm ± 3.1 for D1c; Neck diameter > 20%: 11.5% for D1a, 13.1% for D1b, and 14.8% for D1c	1 (1.6%)	1 (1.6%)	NA	1 (1.6%)	Proximal angioplasty (1)
Savlovskis et al. [13]	<36	>6 mm vs. 0 (Endurant vs. Nellix) Level 1: 0.65 mm/y vs. 0.17 mm/y Level 2: 0.92 mm/y vs. 0.18 mm/y Level 3: 1.0 mm/y vs. 0.22 mm/y	+2.8 mm (Endurant); +2.6 mm (Nellix)	NA	NA	NA	NA
Tsilimparis et al. [4]	<60	0.47 mm/month in the early period 0.10 mm/month in the later period	NA	NA	NA	NA	NA
de Donato et al. [9]	32 (24–50)	Zone A: +0.18 mm; Zone B: −0.32 mm; Zone C: −0.06 mm	0	2 (1.2%)	6 (3.7%)	1 (0.6%)	Proximal angioplasty (1) Graft relining (1)
Kret et al. [6]	21.9 (3.7–63.8)	Early period: +1.34 mm (+5.9%) Late Period: +5.36 mm (+21.7%) Dilation >10% in 55 (62.9%)	0	2 (2.5%)	2 (2.5%)	2 (2.5%)	Surgical conversion (1)
Gargiulo et al. [10]	37.9 (24–103)	All necks increased Significant dilation in D1: 42%, D2: 31%, D3: 32%;	NA	14 (12%)	19 (16%)	8 (7%)	Surgical conversions (7)
Vukovic et al. [5]	<48	2–4 mm Oversize >20%: 0.5 mm/y	Continuous migration, average 6 mm; Oversize >20%: 1.9 mm/y	8 (6.3%)	17 (13.4%)	7 (5.5%)	Surgical conversions (6) Fenestrated cuff (1)
Torsello et al. [14]	<60	25/60 patient (41%) Average: +4.5 mm (+19%) at 15 mm from the lowest RA No modification at renal level	1.9–3.1 mm,	2 (3%)	3 (5%)	2 (3%)	Proximal cuff (1) Chimney graft (1)
Deltomme et al. [8]	<60	At SMA level: no changes only with Endurant At RA level: significant changes with all endografts	0	7 (5.8%)	18 (15%)	7 (5.8%)	Proximal cuff (4), Fenestrated cuff (1), Embolization (2)
Mathlouthi et al. [12]	<60	10 patients (4.2%) Mean dilation: 1 mm	0	NA (3.7%)	43 (18.1%)	0	0
Oliveira et al. [7]	<60	Mean dilation: 3.0 mm (0.4–5.0) Dilation >10%: 241 (52.4%); Dilation >20%: 131 (28.5%); Dilation 1.5%/y	NA	NA	NA	NA	NA
Tran et al. [16]	30.3	32 patients (74.4%); Mean dilation: 3.1 mm (0.99/y)	0	1 (2.3)	8 (18.6%)	1 (2.3%)	NA
Zettervall et al. [17]	<36	Dilation at SMA ^a^: 28 patients (50%); Dilation at RA ^b^: 36 patients (64%)	NA	0	NA	NA	NA
Teter et al. [18]	<52	Dilation at SMA level: 15.5%; Dilation at RA level: + 8.5%	NA	NA	5 (16.6%)	NA	NA
Rastogi et al. [15]	<42	Dilation > 10%: 47.6%; Dilation > 20%: 20.2%	0	2 (2.4%)	NA	1 (1.2%)	NA

^a^ SMA: superior mesenteric artery; ^b^ RA: renal artery.
